# Sentinel lymph node biopsy by transvaginal natural orifice transluminal endoscopic surgery for early-stage endometrial cancer: a systematic literature review

**DOI:** 10.3389/fsurg.2025.1663469

**Published:** 2025-10-23

**Authors:** Déborah Wernly, James Nef, Daniela Huber

**Affiliations:** 1 Department of Gynecology and Obstetrics, Valais Hospital, Sion, Switzerland; 2 Department of Women, Child and Adolescent, Gynecology and Obstetrics, Geneva University Hospitals, Geneva, Switzerland

**Keywords:** sentinel lymph node (SLN) biopsy, vaginal natural orifice transluminal endoscopic surgery (vNOTES), endometrial cancer, sentinel node mapping, minimally invasive surgery, indocyanine green (ICG), carbon nanoparticles

## Abstract

**Introduction:**

Sentinel lymph node (SLN) mapping has become a standard approach for early-stage endometrial malignancies, offering reduced morbidity compared to complete lymphadenectomy. Recently, transvaginal natural orifice transluminal endoscopic surgery (vNOTES) has emerged as a novel minimally invasive technique for SLN biopsy, with potential benefits in early surgical outcomes. This systematic review evaluates current evidence on SLN biopsy performed via vNOTES.

**Material and methods:**

A systematic literature search was conducted in PubMed, Embase, and Web of Science for articles published between January 1, 2014 and January 31, 2025. Studies were included if they reported SLN biopsy by vNOTES in at least 10 patients with early-stage endometrial cancer and provided detailed data on SLN detection. Our primary outcomes focused on SLN detection and failure rates. Secondary objectives included the early operative outcomes. PROSPERO registration number was CRD42024612607.

**Results:**

Seven studies comprising 231 patients were included. The overall bilateral SLN detection rate was 89.2%, with higher detection in the retroperitoneal subgroup (94.3%) compared to the transperitoneal subgroup (81.1%). The overall failure rate was 3.9%. Nodal metastases were reported in 5.6% (10/179) of patients. Intraoperative complications occurred in 4.8% of cases, with bladder injury being the most frequent. The conversion rate to laparoscopy was 6.5%, primarily due to unsuccessful SLN mapping. Postoperative complications occurred in 3% of patients and were mostly minor. Comparisons with conventional laparoscopy showed similar operative times and blood loss, while vNOTES appeared to offer potential advantages in reducing pain and shortening hospital stay.

**Conclusion:**

vNOTES is a promising technique for SLN mapping in early-stage endometrial cancer, demonstrating high detection rates with low complication rates. However, evidence remains limited and heterogeneous, highlighting the need for larger, prospective, and randomized studies to validate long-term oncological safety and define its role in clinical practice.

## 1 Introduction

Endometrial cancer is the most common gynecologic malignancy in developed countries. Incidence and mortality rates continue to rise, largely due to risk factors such as obesity, metabolic syndrome, and increased life expectancy (1–3). Most women are diagnosed at an early stage, with approximately 67% of cases confined to the uterus, resulting in a favorable prognosis (4, 5). Despite the growing adoption of molecular classification, surgical staging remains critical for prognostication and treatment planning. The recent FIGO 2023 staging update reaffirmed the importance of accurate nodal assessment in guiding adjuvant therapy decisions (6). Assessment of lymph node status remains central to surgical staging. While complete lymphadenectomy has shown no survival benefit in early-stage disease, sentinel lymph node (SLN) biopsy provides reliable staging with reduced morbidity (7–9). SLN algorithms and ultrastaging further improve detection of low-volume metastases, guiding adjuvant treatment when needed (3, 5, 10, 11).

For endometrial malignancies, SLN biopsy is mainly performed by conventional laparoscopy or robotic-assisted laparoscopy. Since 2014, following three cases of pelvic lymphadenectomy described by Lee et al. (12), several publications have reported the use of vaginal natural orifice transluminal endoscopic surgery (vNOTES) for surgical lymph node staging in early-stage endometrial cancer. These studies have focused on its feasibility and suggested potential advantages in terms of recovery and postoperative pain (12–14).

This article provides a systematic review of current data on vNOTES for SLN biopsy in early-stage endometrial cancer. We aimed to analyze all published series including 10 or more patients undergoing this approach, focusing on detection rates (bilateral and unilateral), failure rates, and early surgical outcomes, including conversion rates, hybrid access, and intra- and postoperative complications.

## 2 Material and methods

### 2.1 Search strategy and selection criteria

The design of this systematic literature review is consistent with the Preferred Reporting Items for Systematic Reviews and Meta-Analyses (PRISMA) guidelines (15) and is registered in the PROSPERO International Prospective Register of Systematic Reviews (CRD 42024612607).

To identify all the publications reporting data on SLN biopsy in endometrial cancer over the last decade, Pubmed, Embase and Web of Science databases were searched using a combination of keywords including “vNOTES” and “sentinel lymph node” and “endometrial cancer” for all articles published between January 1, 2014 and January 31, 2025.

We included all studies that met the following inclusion criteria: (1) a minimum sample size of ten patients who underwent SLN biopsy via vNOTES for the treatment of early-stage endometrial cancer; (2) detailed data on SLN drainage patterns, specifically distinguishing between bilateral and unilateral drainage; and (3) publication date between January 1, 2014 and January 31, 2025. These criteria were defined to ensure that the included studies provided sufficient patient numbers and relevant clinical data to effectively evaluate the efficacy of SLN biopsy for endometrial cancer by vNOTES.

Publications were selected in a two-step process. First, two reviewers screened and de-duplicated all titles and abstracts identified from the searches independently and blindly based on the predefined inclusion and exclusion criteria. A third reviewer arbitrated in cases of disagreement. Secondly, the same reviewers assessed the full texts of potentially relevant articles independently to determine their eligibility. Any disagreements that arose at this stage were resolved through discussion and consultation with the third reviewer. The reasons for excluding articles were thoroughly documented to ensure the transparency and reproducibility of the review process.

**Figure 1:**
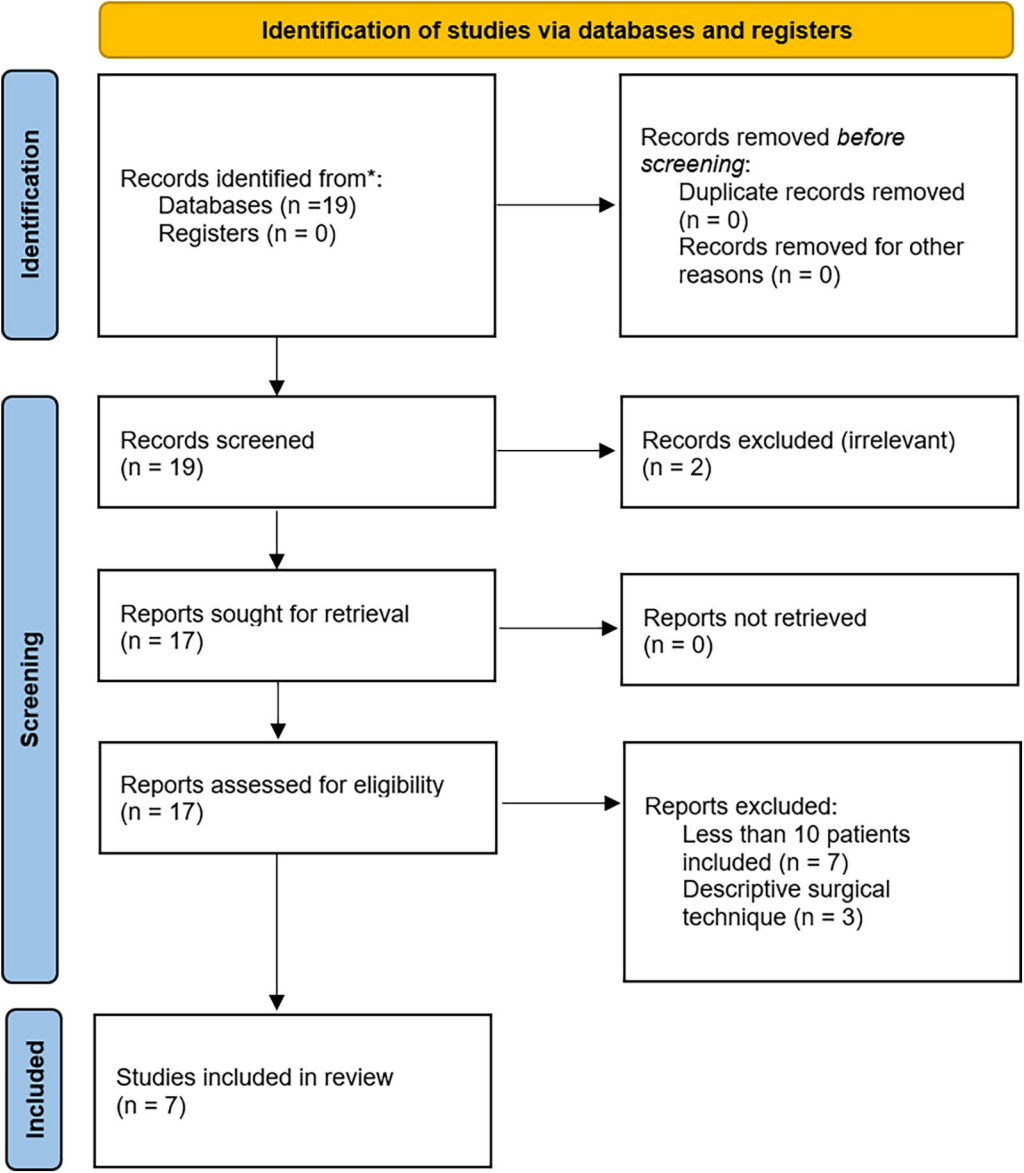
PRISMA 2020 flow diagram for study selection [adapted from Page et al. (15)].

The study design is presented in Figure 1. Ultimately seven studies were retained for analysis (13, 14, 16–20).

### 2.2 Data extraction and analysis

The primary outcome was the SLN detection rates in women with endometrial cancer who underwent vNOTES surgery, categorized into bilateral, unilateral, and failure rates. For each study, the numbers of patients with bilateral, unilateral, or failed detection were extracted. Only the patients who underwent the vNOTES procedures were extracted and analyzed. In addition, the results were stratified by surgical technique, distinguishing between the retroperitoneal and transperitoneal approaches.

Secondary outcomes included operative time, blood loss, conversion rates, and intraoperative and postoperative complications. A subgroup analysis was performed to compare these outcomes by surgical approach, distinguishing between retroperitoneal and transperitoneal access. Studies lacking outcome details were excluded from the respective analyses, and missing data were not imputed.

Risk of bias and study quality were assessed using the Mixed Methods Appraisal Tool (MMAT). Quality assessment did not influence study inclusion, which was determined exclusively by the predefined eligibility criteria, but MMAT ratings were considered in the interpretation of findings. Two reviewers independently assessed each included article for risk of bias, with disagreements resolved by consensus.

## 3 Results

Of the seven included cohorts, four were prospective and three were retrospective. Indocyanine green (ICG) was used as a tracer in six studies and carbon nanoparticles (CNP) in two. Four studies included high-risk histologies. Only two studies were multicentre (Table 1).

**Table 1:** Characteristics of the included studies.

Study	Number of patients	Methodology	Number of centers	Tracer	High-risk carcinoma
	(*n*)		(*n*)		
Wang et al. (18)	23	R	Mono	CNP	N
Lee et al. (16)	10	P	Mono	ICG	N
Deng et al. (14)	57	P	Multi (2)	CNP/ICG	Y
Baekelandt et al. (19)	64	P	Multi (4)	ICG	N
Comba et al. (13)	19	R	Mono	ICG	Y
Simsek et al. (20)	24	R	Mono	ICG	Y
Huber et al. (17)	34	P	Mono	ICG	Y

High-risk carcinoma: non-endometrioid histology, grade 3, substantial lymphovascular space invasion (LVSI).

ICG, Indocyanine green; CNP, carbon nanoparticle; R, retrospective; P, prospective; Y, yes; *N*, no.

Data from 231 patients who underwent SLN biopsy for early-stage endometrial cancer between 2016 and 2024 were analyzed across seven studies. The mean age was 60 years with a mean BMI 26.8 kg/m^2^. A transperitoneal approach was performed in 90 patients and a retroperitoneal approach in 141 cases. A single midline anterior vaginal incision was used to access the pelvic retroperitoneal space in 32 cases, while two bilateral incisions in the lateral vaginal fornices were used in 109 cases.

The overall bilateral SLN detection rate was 206/231 (89.2%), with 73/90 (81.1%) in the transperitoneal group and 133/141 (94.3%) in the retroperitoneal group. SLN detection failed in 9/231 patients (3.9%): 6/90 (6.7%) transperitoneal and 3/141 (2.1%) retroperitoneal (Table 2). Empty packet dissections were reported in one study (3/19 patients) (16). Four studies reported nodal metastases: 10/179 patients (5.6%) (14, 17, 19, 20).

**Table 2:** SLN detection and failure rates.

Study	SLN approach	Bilateral identification	Unilateral identification	Detection failure
		(%)	(%)	(%)
Wang et al. (18)	TP	20/23 (87.0)	2/23 (8.7)	1/23 (4.3)
Lee et al. (16)	TP	6/10 (60.0)	2/10 (20.0)	2/10 (20.0)
Deng et al. (14)	TP	47/57 (82.5)	7/57 (12.3)	3/57 (5.3)
Baekelandt et al. (19)	RP (L&A)	62/64 (96.9)	2/64 (3.1)	0/64 (0.0)
Comba et al. (13)	RP (L)	18/19 (94.7)	0/19 (0.0)	1/19 (5.3)
Simsek et al. (20)	RP (L)	22/24 (91.6)	1/24 (4.2)	1/24 (4.2)
Huber et al. (17)	RP (L)	31/34 (91.2)	2/34 (5.9)	1/34 (2.9)
Overall	TP&RP	206/231 (89.2)	11/90 (6.9)	9/231 (3.9)
	TP	73/90 (81.1)	11/90 (12.2)	6/90 (6.7)
	RP	133/141 (94.3)	5/141 (3.6)	3/141 (2.1)

SLN, sentinel lymph node; TP, transperitoneal; RP, retroperitoneal; L, lateral; A, anterior.

Intraoperative complications occurred in 11/231 patients (4.8%), most commonly bladder injury (4/231, 1.7%). Other events were bleeding or vascular injury (2/231, 0.9%), obturator nerve neuropraxy (1/231, 0.4%), peritoneal defect (1/231, 0.4%), and atrial fibrillation (1/231, 0.4%) (Table 3). Two patients required ICU admission because of comorbidities. The conversion rate to conventional laparoscopy was 15/231 (6.5%), mainly for unsuccessful SLN mapping (12/231, 5.2%). Additional reasons were bleeding (2/231, 0.9%) and bladder injury (1/231, 0.4%). No conversion to laparotomy was reported. The mean operative time was 141 min, and the mean blood loss of was 80 ml.

**Table 3:** Intra- and postoperative complications.

Study	Intraoperative complication	Conversion to laparoscopy	Postoperative complications
	*n*/*N* (%)	*n*/*N* (%)	*n*/*N* (%)
Wang et al. (18)	0/ 23 (0.0)	1/23 (4.3)	1/23 (4.3)^a^
Lee et al. (16)	1/10 (8.3)^b^	1/10 (8.3)	0/10 (0.0)
Deng et al. (14)	0/57 (0.0)	10 (17.5)	0/57 (0.0)
Baekelandt et al. (19)	8/64 (12.5)^c^	1/64 (1.6)	4/64 (6.3)^d^
Comba et al. (13)	2/19 (10.5)^e^	1/19 (5.3)	1/19 (5.3)^f^
Simsek et al. (20)	0/24 (0.0)	0/24 (0.0)	0/24 (0.0)
Huber et al. (17)	N/A	1/34 (2.9)	N/A

a Poor healing of the vaginal stump.

b Bladder lesion.

c Cystotomies (3), Adductor paresis (1), Left obturator vein cut (1), Peritoneal defect (1), Atrial fibrillation during surgery (1), Left obturator space bleeding (1).

d Paresis of the adductor muscles of the hip (1), femoral deep vein thrombosis (1), Postoperative bleeding infundibulopelvic ligament (1), and vaginal vault hematoma (1).

e ICU admissions (2): obesity and HTA/diabetes.

f Vaginal hematoma (1).

Postoperative complications were observed in 6/231 patients (2.6%): bleeding (1/231, 0.4%), hip adductor paresis (1/231, 0.4%), femoral DVT (1/231, 0.4%), poor vaginal stump healing (1/231, 0.4%), and vaginal hematomas (2/231, 0.9%). One patient had both DVT and hematoma (Table 3).

Only three studies reported follow-up (range 6–29 months): Comba et al. 22.4 ± 8.4 months, Lee et al. 28.6 months, and Wang et al. 6 months. No recurrences were observed.

MMAT appraisal indicated overall good methodological quality, though patient selection processes were sometimes insufficiently described (Table 4).

**Table 4:** Ratings for mixed methods appraisal tools (MMAT).

Study	Quantitative non-randomized							Quantitative descriptive				
	S1	S2	3.1	3.2	3.3	3.4	3.5	4.1	4.2	4.3	4.4	4.5
Comba et al. (13)	Y	Y	Y	Y	Y	C	Y					
Deng et al. (14)	Y	Y	Y	Y	Y	Y	Y					
Huber et al. (17)	Y	Y						Y	Y	Y	Y	Y
Lee et al. (16)	Y	Y						Y	N	Y	C	Y
Simsek et al. (20)	Y	Y						Y	N	Y	Y	Y
Wang et al. (18)	Y	Y						Y	Y	Y	N	N
Baekelandt et al. (19)	Y	Y						Y	Y	Y	Y	Y

Y, yes; N, no; C, can't tell.

## 4 Discussion

This systematic review shows high bilateral detection and low complication rates for SLN biopsy via vNOTES in early-stage endometrial cancer. These rates are in line with previously reported data for minimally invasive surgery. The SHREC study (9) reported a bilateral SLN mapping rate of up to 95% using a robotic approach. In comparison, the Senti-Endo study (21) achieved an 89% detection rate with either laparoscopic or open surgical techniques. Subsequent studies have reported bilateral detection rates ranging from 65%–88% (22). By comparison, the prospective FIRES study (23), which utilized a robotic approach, reported a lower bilateral detection rate of 52%, likely attributable to limited surgeon experience.

All studies included in our review performed sentinel lymph node mapping using cervical injection, which is consistent with current evidence suggesting that this is probably the most reliable injection site for uterine malignancies. Cervical injection offers reproducible high bilateral detection rates and is now widely regarded as the preferred approach (24).

The risk of empty packets is reported only in one study (16), affecting 3 out of 10 patients. Although the literature on this topic is limited, the reported incidence appears to range between 5% and 9% (25, 26). This risk seems to decrease with increasing surgical experience (25, 26). The choice of tracer significantly influences the occurrence of empty packets. Minareci et al. (25) reported no empty packets when using CNP, in contrast to ICG, which may leak from injured or dilated lymphatic vessels into surrounding fat tissue, potentially mimicking lymph nodes. Two studies report data on the location of SLNs harvested by vNOTES, both using a retroperitoneal approach and with similar results. The SLNs identified by vNOTES are predominantly located in the obturator region (80%) (17, 20). In comparison, the FIRES trial (23) found SLNs most frequently in the external iliac region (38%), followed by the obturator (25%), inframesenteric para-aortic (14%), internal iliac (10%), common iliac (8%), presacral (3%), infrarenal para-aortic (1%), and other areas (including the parametrium) (1%). SLNs tend to be mapped symmetrically, with rates ranging from 71%–79% (17, 20, 27). A recent study (28) shows that 49.1% of positive SLNs are located in the proximal obturator region. Therefore, the authors suggest that if SLN detection is unsuccessful, lymphadenectomy could be limited to the proximal obturator and interiliac areas. These regions are easily explored with the vNOTES approach, allowing for targeted pelvic lymphadenectomy if necessary (29, 30).

Nodal metastasis was identified in 5.6% (10 out of 179) of cases. Large cohort studies have documented SLN metastasis rates in early stage endometrial cancer ranging from 6%–10% (5, 28, 31). These cases of nodal metastasis came from four studies (14, 17, 19, 20), that included a high proportion of patients with low-risk histology: 125 out of 179 patients (69.8%) were FIGO stage IA, 115 (64.2%) had grade 1 tumors, and in the two studies (14, 20) that reported myometrial invasion, 66 out of 81 patients (81.5%) had superficial infiltration.

The perioperative and postoperative complication rates are consistent with published data for other minimally invasive approaches to SLN biopsy. In the FIRES trial (23), the overall adverse event rate was 9%, including 6% serious adverse events. The intraoperative complication rate was 1%, including three bowel lesions and one ureteral injury; the postoperative complication rate (8%) was mostly neurological or respiratory. The SHREC (9) and SENTOR (10) trials reported both a 3% intraoperative complication rate, mostly vascular injury, and a 32% and 26% postoperative complication rate respectively, the majority of which were minor (grade 1–2).

In this systematic review, bladder injury was the most common intraoperative complication, comparable to the rates reported for both laparoscopic and open surgeries performed for early-stage endometrial cancer. A meta-analysis of seven randomized controlled trials (RCTs) (32) including 3,342 women undergoing surgical treatment for early-stage endometrial cancer found no statistically significant difference in the risk of bladder injury between laparoscopy and laparotomy, estimating an overall incidence of approximatively 1%. In our study, bladder injury was more frequent with retroperitoneal access, an increase likely due to the bilateral para-vesical dissection and the surgeon's learning curve.

Three studies included in our systematic review (13, 14, 18) provide comparative data with conventional laparoscopy. Operative time and blood loss are similar between the two surgical techniques. In addition, patients undergoing vNOTES appear to experience less postoperative pain and benefit from a shorter hospital stay which appear to be comparable to other larger non-oncological vNOTES cohorts (33, 34).

Two surgical approaches are reported for SLN harvest by vNOTES: transperitoneal and retroperitoneal. The transperitoneal approach is performed after hysterectomy, with the benefit that tumor removal precedes lymph node staging and the risk of retroperitoneal malignant contamination is reduced. However, this sequence does not allow for the reinjection of ICG if needed, and tracer leakage may occur. That might explain the lower rates of bilateral detection with the transperitoneal approach, as the tracer reinjection has been shown to increase the SLN detection rate from 82%–95% (9). Two studies have used CNP as a tracer (14, 18) instead of ICG to address this issue.

Transperitoneal access appears to be very similar to conventional laparoscopy and easier to adopt by surgeons experienced in SLN mapping using standard minimally invasive techniques (17, 18). Nevertheless, the exposure of the caudal obturator space is limited by the intraperitoneal placement of the transvaginal platform compared to SLN biopsy by conventional laparoscopy or robotic surgery.

The retroperitoneal approach allows for cervical ICG reinjection (35). While this is a major advantage as it increases the detection rate, the fact that the SLN is harvested prior to the tumor removal also raises concerns about the potential dissemination of malignant cells within the retroperitoneum. To reduce the risk of tumor spillage, protective maneuvers such as cervical cerclage or closure of the external cervical os with sutures may be performed (14).

The vNOTES approach to SLN biopsy for endometrial cancer offers several potential advantages over traditional minimally invasive surgery. Unlike conventional endoscopic approaches, retroperitoneal vNOTES SLN biopsy does not require Trendelenburg positioning, thereby reducing hemodynamic and respiratory strain. This benefit is especially valuable in obese patients, in elderly individuals or those with significant cardiopulmonary comorbidity who may not tolerate prolonged Trendelenburg positioning. In addition, the vNOTES approach bypasses challenges in peritoneal access due to abdominal wall thickness or extensive adhesions from prior abdominal surgeries, further highlighting its potential role in carefully selected clinical scenarios.

This systematic review summarizes published data on SLN biopsy in endometrial cancer using the vNOTES approach. Although still an emerging technique, it appears to be feasible and with reassuring early surgical outcomes.

Several limitations must be acknowledged. First, most studies were single-center and non-randomized, raising the possibility of selection bias, since patients undergoing vNOTES may represent a favorable subgroup with lower BMI, fewer comorbidities, or lower disease stage. Second, tracer protocols, surgical access (retroperitoneal vs. transperitoneal), and perioperative outcome reporting varied widely, limiting the comparability of results. Finally, follow-up was short or absent in most studies, preventing any conclusions regarding long-term oncologic safety. Future research should prioritize multicenter prospective trials with standardized oncological endpoints (recurrence, disease-free survival), cost-effectiveness analyses, and patient-reported outcomes to better define the role of vNOTES.

Despite these limitations, the current evidence suggests that vNOTES could expand the armamentarium of minimally invasive techniques for SLN biopsy. This review demonstrates its feasibility and favorable early surgical outcomes, but its adoption should remain limited to centers with expertise, within prospective studies or registries. Future multicenter randomized trials with standardized reporting are needed to confirm these preliminary results and clarify the place of vNOTES in the surgical management of endometrial cancer.

## 5 Conclusion

vNOTES is a promising technique for SLN biopsy in endometrial cancer. It is emerging as an alternative option to conventional laparoscopic and robotic approaches, particularly in obese patients, in those with comorbidities that limit Trendelenburg positioning, or in those with prior abdominal scarring and suspected abdominal wall adhesions. Current evidence is limited, heterogeneous, and largely based on small single-center studies. Larger prospective and randomized trials are required to confirm detection performance, assess long-term oncologic safety, and define its role before widespread clinical adoption.
